# The phospho-barcode of RIPK1: complementarity or redundancy?

**DOI:** 10.1080/23723556.2020.1776085

**Published:** 2020-07-16

**Authors:** Wenxian Wu, Björn Stork

**Affiliations:** Institute of Molecular Medicine I, Medical Faculty, Heinrich Heine University Düsseldorf, Düsseldorf, Germany

**Keywords:** Autophagy, necroptosis, RIPK1, TNF, Ulk1

## Abstract

Receptor interacting serine/threonine kinase 1 (RIPK1) is the central mediator of tumor necrosis factor (TNF) signaling. It regulates both pro-survival/pro-inflammatory and cell death pathways. In order to fulfill this complex regulation, RIPK1 is regulated by several post-translational modifications, including ubiquitination, acetylation, and phosphorylation. In our recent work, we show that the unc-51-like autophagy activating kinase 1 (ULK1) phosphorylates RIPK1 at Ser357 and thus blocks TNF-induced cell death.

Receptor interacting serine/threonine kinase 1 (RIPK1) is the central mediator of the signaling cascades downstream of the tumor necrosis factor (TNF) receptor superfamily member 1A (TNFRSF1A, also known as TNFR1). RIPK1 is composed of an N-terminal kinase domain, an intermediate domain containing a RIP homotypic interaction motif (RHIM), and a C-terminal death domain (DD). Upon TNF stimulation, a TNFR1-associated “complex I” is assembled that consists of RIPK1, TNFRSF1A associated via death domain (TRADD), TNF receptor-associated factor 2/5 (TRAF2/5), baculoviral IAP repeat-containing 2/3 (BIRC2/3; also known as cellular inhibitors of apoptosis proteins 1/2, cIAP1/2), and the linear ubiquitin chain assembly complex (LUBAC). cIAP- and LUBAC-generated ubiquitin chains then establish the platform for the recruitment of further protein complexes such as TAB2-TAB3-MAP3K7/TAK1 and IKBKG/NEMO-CHUK/IKKα-IKBKB/IKKβ, which mediate activation of mitogen-activated protein kinase (MAPK) and nuclear factor kappa-light-chain-enhancer of activated B (NF-κB) signaling pathways. Upon dissociation of RIPK1 from complex I (either caused by deubiquitination or by inhibited ubiquitination), different cytosolic RIPK1-dependent complexes are assembled that mediate apoptotic (complex IIa, complex IIb) or necroptotic cell death (necrosome). Generally, kinase-active RIPK1 supports apoptosis and necroptosis, whereas kinase-inactive or scaffolding functions of RIPK1 inhibit these events. If deubiquitinated RIPK1 and inactivated caspase 8 are present, necroptosis is induced. Kinase-active RIPK1 and receptor interacting serine/threonine kinase 3 (RIPK3) are required for the assembly of the necrosome, an amyloid-like signaling complex. The RIPK3 oligomerization then leads to RIPK3 autophosphorylation and transphosphorylation of the mixed lineage kinase domain like pseudokinase (MLKL). Ultimately, phosphorylated MLKL translocates to the plasma membrane and induces pore formation.

In recent years, a complex RIPK1 “phospho-barcode” has been established (Figure 1). In 2008, Degterev et al. reported the identification of several autophosphorylation sites.^1^ Of these sites, Ser14/15, Ser20, Ser161, and Ser166 were phosphorylated only in samples subjected to the kinase reactions (Figure 1). Other residues of RIPK1 (Ser6, Ser25, Ser303, Ser320, Ser330/331, and Ser333) were phosphorylated even in the absence of the kinase reaction, indicating that these sites were phosphorylated in the cells before immunopurification.^1^ Since then, several RIPK1-phosphorylating kinases have been reported. In 2015, Dondelinger et al. demonstrated that component of inhibitor of nuclear factor kappa B kinase complex (CHUK, also known as the inhibitor of κB [IκB] kinase α, IKKα) and inhibitor of nuclear factor kappa B kinase subunit beta (IKBKB, also known as IKKβ) directly phosphorylate RIPK1 within complex I.^2^ The identified sites include Ser25 and Ser166 in the kinase domain, and Ser296, Ser331, and Ser416 in the intermediate domain (Figure 1).^2^ In 2019, the same group showed that IKKα/IKKβ-dependent phosphorylation of RIPK1 at Ser25 directly inhibits RIPK1 kinase activity and prevents TNF-mediated RIPK1-dependent cell death.^3^ Additionally, cellular phosphorylations sensitive to an IKK inhibitor have been confirmed for Ser330/331 and Ser416.^4^ Three groups independently reported the phosphorylation of RIPK1 by MAPK activated protein kinase 2 (MAPKAPK2, also known as MK2), which acts downstream of the TAB2-TAB3-MAP3K7/TAK1 complex.^5-7^ In the case of MK2, the major phospho-acceptor sites are Ser320 and Ser335, respectively (Figure 1). It appears that primarily the cytosolic pool of RIPK1 becomes phosphorylated, but this phosphorylation still allows recruitment of RIPK1 to complex I. Similar to the IKKα/IKKβ-dependent phosphorylation, MK2-dependent RIPK1 phosphorylation suppresses its integration into cytoplasmic cytotoxic complexes and RIPK1-dependent cell death pathways. Finally, it has also been shown that TANK binding kinase 1 (TBK1) and inhibitor of nuclear factor kappa B kinase subunit epsilon (IKBKE, also known as IKKε) phosphorylate multiple sites of RIPK1 (Figure 1).^8,9^ These phosphorylations were reported to occur within complex I and independently of IKKα, IKKβ, and MK2, but again prevent RIPK1 autophosphorylation and assembly of complex II.^8^

**Figure 1.**
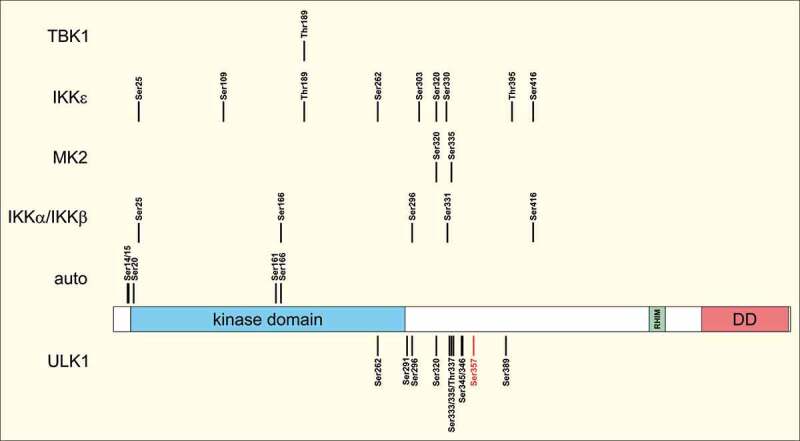
RIPK1 phosphorylation sites. Receptor-interacting serine/threonine kinase 1 (RIPK1) consists of an N-terminal kinase domain, an intermediate domain harboring a RIP homotypic interaction motif (RHIM) and a C-terminal death domain (DD). The scheme depicts the phosphorylations mediated by RIPK1 (autophosphorylation), component of inhibitor of nuclear factor kappa B kinase complex (CHUK, also known as the inhibitor of κB [IκB] kinase α, IKKα) and inhibitor of nuclear factor kappa B kinase subunit beta (IKBKB, also known as IKKβ), MAPK activated protein kinase 2 (MAPKAPK2, also known as MK2), inhibitor of nuclear factor kappa B kinase subunit epsilon (IKBKE, also known as IKKε), TANK binding kinase 1 (TBK1), and unc-51-like autophagy activating kinase 1 (ULK1). Ser357 (highlighted in red) represents a major ULK1-dependent phospho-acceptor site. The numbering refers to amino acid positions in human RIPK1 (UniProt ID Q13546).

In our study, we originally aimed at investigating the crosstalk between the unc-51-like autophagy activating kinase 1 (ULK1) and the cell death-regulating kinase RIPK1.^10^ Although RIPK1 has been connected to autophagy signaling in general, a direct crosstalk between the two executioner kinases of these cellular stress responses has not been described so far. In our in vitro kinase assays followed by mass spectrometry, we identified several ULK1-dependent phospho-acceptor sites in RIPK1, including Ser262, Ser291, Ser296, Ser320, Ser333, Ser335, Thr337, Ser345, Ser346, Ser357, and Ser389 (Figure 1).^10^ Of these, Ser357 gained our major interest, since it was the only residue whose exchange for alanine clearly reduced the intensity of the phosphorylation signal obtained in the in vitro kinase assay. Of note, also ULK1-dependent phosphorylation of RIPK1 prevented execution of cell death pathways, similar to the kinases described above. We expressed the S357A variant of RIPK1 in *Ripk1* KO MEFs and observed that RIPK1 autophosphorylation at Ser166 and phosphorylation of the necroptosis effector protein MLKL at Ser345 were increased compared to cells expressing wild-type RIKP1. TSZ [TNF + Smac mimetic + zVAD]-induced cell death was also enhanced in RIPK1 S357A-expressing cells, and was not sensitive to siRNA-mediated ULK1 knockdown. Finally, we also observed that complex IIb/necrosome was stabilized in RIPK1 S357A-expressing cells upon TSZ treatment, since we detected increased RIPK1 Ser166 phosphorylation, increased generation of the p43 fragment of caspase 8, and increased association of RIPK3. Although the phosphorylations described above have in common that they inhibit the cell death-inducing arm of RIPK1 signaling, they differ in the location where these phosphorylations take place, i.e. within the TNFR1-associated complex I or in the cytosol. With regard to ULK1, we observed that ULK1 also associates with complex I, but that Ser357 phosphorylation is not evident for complex I-associated RIPK1. Association with complex I appeared slightly increased for RIPK1 itself in cells expressing ULK1. In contrast, in post-TNFR1-immunopurified lysates, Ser357 was readily detectable and RIPK1 autophosphorylation was reduced. Accordingly, we propose that ULK1 can stabilize RIPK1 in complex I and negatively regulates RIPK1 activity in the cytosol. Nevertheless, the complementarity or redundancy of the above-mentioned phosphorylation events awaits further clarification and might be affected by cell type-specific aspects or the type of RIPK1-engaging stimulus. For some of the phospho-acceptor sites, several kinases have been proposed, and future studies will have to reveal their relative contribution to RIPK1-dependent signaling. It has been proposed that the assembly of complex II is initiated from complex I-derived RIPK1, which serves as seeding point and is then augmented by cytosolic RIPK1.^6^ Presumably, both the seeding and the further recruitment are fine-tuned by this diverse set of upstream kinases.
